# *Candidatus* Neoehrlichia mikurensis Infection in Patient with Antecedent Hematologic Neoplasm, Spain^1^

**DOI:** 10.3201/eid2908.230428

**Published:** 2023-08

**Authors:** Paola González-Carmona, Aránzazu Portillo, Cristina Cervera-Acedo, Daniel González-Fernández, José A. Oteo

**Affiliations:** Hospital de Jarrio, Asturias, Spain (P. González-Carmona, D. González-Fernández);; Hospital Universitario San Pedro-CIBIR, La Rioja, Spain (A. Portillo, C. Cervera-Acedo, J.A. Oteo)

**Keywords:** neoehrlichiosis, *Candidatus* Neoehrlichia mikurensis, ticks, *Ixodes ricinus*, lymphoma, hematologic neoplasm, bacteria, parasites, vector-borne infections, zoonoses, Spain

## Abstract

We report a confirmed case of *Candidatus* Neoehrlichia mikurensis infection in a woman in Spain who had a previous hematologic malignancy. *Candidatus* N. mikurensis infections should be especially suspected in immunocompromised patients who exhibit persistent fever and venous thrombosis, particularly if they live in environments where ticks are prevalent.

*Candidatus* Neoehrlichia mikurensis is an α1-proteobacterium (family Anaplasmataceae) transmitted by *Ixodes* spp. ticks. Although previously described in ticks and mammals in Europe and Asia, the species name was derived from a report in 2004 from Mikura Island, Japan, where the bacterium was found in endothelial cells from rat (*Rattus norvegicus*) spleens and in *Ixodes ovatus* ticks (*1*). In 2010, *Candidatus* N. mikurensis was identified as a human pathogen in Sweden (*2*). Since then, several case series and individual cases of patients with *Candidatus* N. mikurensis infections have been described, mainly in persons who were immunosuppressed because of hematologic neoplasms, splenectomies, or immunosuppressive drug treatment (*3*–*9*). However, *Candidatus* N. mikurensis can cause disease (neoehrlichiosis) in immunocompetent persons or cause asymptomatic infections (*10*,*11*). In 2019, *Candidatus* N. mikurensis was cultured in tick cell lines and infection was transferred to human endothelial cells derived from skin microvasculature and pulmonary arteries, demonstrating endothelial cell tropism. Tropism partly explains the clinical spectrum caused by the bacterium, usually consisting of persistent and recurrent fever and thrombosis and vasculitis with or without erysipelas-like skin lesions (*12*). In Spain, *Candidatus* N. mikurensis was found in *Ixodes ricinus* ticks removed from cows in 2013, but the bacterium was not detected in humans (*13*). We describe a case of *Candidatus* N. mikurensis infection in an immunocompromised patient from Asturias in northern Spain.

## The Study

In September 2020, stage IV-B germinal center diffuse large B-cell lymphoma was diagnosed in a splenectomy specimen from a 68-year-old woman. She completed first-line treatment with rituximab plus cyclophosphamide, doxorubicin, vincristine, and prednisone and achieved complete remission. On June 21, 2021 (≈5 months after lymphoma treatment had ended), she experienced arthromyalgia, anorexia, night sweats, and vespertine fever. Her family physician began treatment with metamizole and cefuroxime at usual doses because of urine sediment alterations. Several days later, deep vein thrombosis developed in her right leg. Because of her previous malignancy and treatment, she was attended at her hospital’s hematology service. She was slightly anemic (hemoglobin 11.7 g/dL, reference range 12–16 g/dL) and had leukopenia (2.28 × 10^3^ leukocytes/µL, reference range 4–14 × 10^3^ leukocytes/µL) and a low neutrophil count (0.4 × 10^3^ neutrophils/µL, reference range 1.8–8.5 × 10^3^ neutrophils/µL). C-reactive protein level was elevated (62 mg/L, reference range <10 mg/L), hyponatremia was present (133 mmol Na/L, reference range 135–145 mmol Na/L), and high levels of ferritin (536 µg /L, reference range 20–200 µg/L) and β2 microglobulin (8.50 mg/L, reference range 0.8–2.4 mg/L) were observed. Other measured hematologic and biochemical parameters, including procalcitonin, were within reference ranges. Other analyses, such as antinuclear antibody testing, blood and urine cultures, and serologic assays against *Coxiella burnetii*, herpes virus, cytomegalovirus, and Epstein-Barr virus, did not indicate acute infection. A chest radiograph and computed tomography scan and an abdominal ultrasound did not reveal pertinent abnormalities. Recurrence of lymphoma was suspected, and a positron emission tomography/computed tomography scan showed diffuse and homogeneous bone marrow hypermetabolism without evidence of neoplastic activity at other levels. 

Empirical treatment was begun with piperacillin/tazobactam and granulocyte colony stimulating factor at conventional doses; 1 week later, the patient had recovered from leukopenia, but fever persisted. A bone marrow biopsy, which did not show neoplastic infiltration or alterations in hematopoietic cells, was performed and processed for different microbiologic tests. A possible tick-related infection was suspected because the patient lived in an area endemic for Lyme disease and other tickborne diseases. The patient recalled having suffered a tick bite 20 days before onset of symptoms. A bone marrow DNA extract and serum sample collected during the acute infection phase (August 2021) were sent to the Special Pathogens Laboratory, Center for Rickettsioses and Arthropod-Borne Diseases, at San Pedro University Hospital–Center for Biomedical Research of La Rioja in Logroño, Spain, to screen for *Candidatus* N. mikurensis by using PCR and *Anaplasma phagocytophilum* by using PCR and immunofluorescence assays. 

We performed PCR targeting the panbacterial 16S rRNA gene, fragments of 16S rRNA gene from Anaplasmataceae (designated as 16S rRNA-EHR), *groEL* from *Candidatus* N. mikurensis, and *msp2* from *A. phagocytophilum* (Table 1). We detected PCR amplicons of the expected sizes for *groEL* and panbacteria and family-specific 16S rRNA in bone marrow and acute phase serum samples; nucleotide sequences corresponded to *Candidatus* N. mikurensis. The *groEL* amplicon (1,232 bp) showed the highest (99.3%) sequence similarity with that of *Candidatus* N. mikurensis from a wild rodent (*Microtus agrestis*) from Siberia in Russia (GenBank accession no. MN701626) but differed from other highly conserved sequences from Siberia and the Far East; the sequence was 98.8% identical to *Candidatus* N. mikurensis found in *Ixodes ricinus* ticks from Spain (*13*) (Table 2). We constructed a phylogenetic tree for *groEL* sequences by using the maximum likelihood method (Figure). We found no differences for the 16S rRNA-EHR sequence (306 bp). The panbacteria 16S rRNA sequence (available upon request from the authors) showed 3–27 mismatches with the 16S rRNA from *Candidatus* N. mikurensis. We did not detect *A*. *phagocytophilum* by PCR in the acute samples. We deposited nucleotide sequences of *groEL* and 16S rRNA genes generated in this study in GenBank under accession nos. OQ579033 (*groEL*) and OQ581737 (16S rRNA).

**Table 1:** PCR primer pairs and conditions used in study of *Candidatus* Neoehrlichia mikurensis infection in patient with antecedent hematologic neoplasm, Spain*

Organisms	Target gene	Primer name	Primer sequence, 5′ → 3′	Amplicon size	Tm, °C
Bacteria	16S rRNA	fD1	AGAGTTTGATCCTGGCTCAG	1,500 bp	60
		rP2	ACGGCTACCTTGTTACGACTT		
Anaplasmataceae†	16S rRNA-EHR	EHR16SD	GGTACCYACAGAAGAAGTCC	345 bp	55
		EHR16SR	TAGCACTCATCGTTTACAGC		
*Anaplasma phagocytophilum*	*msp2*	msp2–3F	CCAGCGTTTAGCAAGATAAGAG	334 bp	56
		msp2–3R	GCCCAGTAACAACATCATAAGC		
*Candidatus* N. mikurensis	*groEL*, 1st run	Ne-groEL-F	GAAGTATAGTTTAGTATTTTTGTC	1,275 bp	49
		Ne-groEL-R	TTAACTTCTACTTCGCTTG		
	*groEL*, 2nd run	Ne-groEL-F	GAAGTATAGTTTAGTATTTTTGTC	510 bp	49
		Ne-groEL_ne-1	ACATCACGTTTCATAGAA		
	*groEL*, 2nd run	Ne-groEL_ne-2	AAAGGAATTAGTATTAGAATCTTT	569 bp	49
		Ne-groEL_ne-4	CTTCCATTTTAACTGCTAA		
	*groEL*, 2nd run	Ne-groEL_ne-3	AATATAGCAAGATCAGGTAGAC	461 bp	49
		Ne-groEL-R	TTAACTTCTACTTCGCTTG		

*Tm, melting temperature. †Includes *Anaplasma*, *Ehrlichia*, and *Candidatus* Neoehrlichia spp. 16S rRNA-EHR refers to the 16S rRNA sequence from the Anaplasmataceae family members, whereas 16S rRNA refers to the panbacterial 16S rRNA sequence.

**Table 2:** Sequence analyses of targeted genes after PCR of DNA from different clinical samples in study of *Candidatus* Neoehrlichia mikurensis infection in patient with antecedent hematologic neoplasm, Spain*

Disease stage	Clinical sample	PCR target genes		
		Panbacteria 16S rRNA	16S rRNA-EHR†	*groEL*‡
Acute	Bone marrow	98% (13,35/1,362) to 99.8% (1,359/1,362),§ CP054597	100% (306/306), CP054597	99.3% (1,224/1,233), MN701626; 98.9% (1,218/1,232), CP054597
	Serum	95.4% (753/789),§ CP054597	ND	99.3% (1,224/1,233), MN701626; 98.9% (1,218/1,232), CP054597
Convalescent, 4th mo	EDTA blood	98% (1,305/1,332), CP054597	ND	ND
	Serum	ND	ND	ND
Convalescent, 6th mo	EDTA blood	ND	ND	ND
	Serum	ND	ND	ND

*Values are highest % identity (identical base pairs/total base pairs), followed by GenBank accession number. ND, not detected. †16S rRNA-EHR refers to the 16S rRNA sequence from the Anaplasmataceae family, which is different from the PCR-amplified panbacteria 16S rRNA sequence. ‡ Percentage of identity with *groEL* *Candidatus* N. mikurensis gene from a reference patient in Sweden (GenBank accession no. CP054597) was lower than that of *groEL Candidatus* N. mikurensis isolate Omsk-41_Micagr from a small mammal in Siberia, Russia (GenBank accession no. MN701626). §Sequence with degenerate bases or obtained from only 1 DNA strand (insufficient sample).

**Figure:**
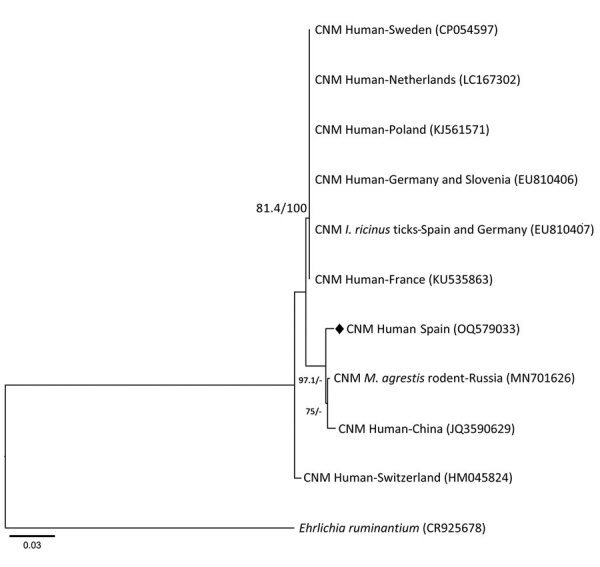
Phylogenetic analysis of *groEL* gene from *Candidatus* Neoehrlichia mikurensis infecting a patient with antecedent hematologic neoplasm, Spain. Phylogenetic tree was generated to compare 809 bp fragments of the 60-kDa heat shock protein gene *groEL* from *Candidatus* Neoehrlichia mikurensis by using IQ-tree software version 2.2.0 (http://www.iqtree.org), maximum-likelihood method, and substitution model consisting of 3-parameter model 2 plus empirical base frequencies with rate heterogeneity allowing for a proportion of invariable sites. Values are approximate likelihood ratio test/bootstrap percentages, indicating topologic branch support for maximum-likelihood analysis with 1,000 replicates; values >75% define high stability. Diamond indicates nucleotide sequence of *Candidatus* N. mikurensis *groEL* gene fragment obtained in this study. *Ehrlichia ruminantium* (Anaplasmataceae family) *groEL* sequence was used as the outgroup. GenBank accession numbers are in parentheses. CNM, *Candidatus* N. mikurensis; *I*. *ricinus*, *Ixodes ricinus*; *M*. *agrestis*, *Microtus agrestis*. Scale bar indicates nucleotide substitutions per site.

On the basis of PCR results, the patient was treated with doxycycline (100 mg 2×/d for 3 wk), and fever disappeared after 72 hours. Neutropenia was attributed to the intake of metamizole for symptom control. However, another case of doxycycline-treated *Candidatus* N. mikurensis infection associated with neutropenia has been reported (*8*). EDTA-anticoagulated blood and serum specimens were collected 4 (December 2021) and 6 (February 2022) months after onset of the acute infection phase, and we screened for *Candidatus* N. mikurensis at the Center for Rickettsioses and Arthropod-Borne Diseases, as previously described. We detected *Candidatus* N. mikurensis DNA in blood collected at 4 months but not in serum. The patient was healthy and blood test results did not show abnormalities at that time. Follow-up PCR of specimens collected at 6 months yielded negative results (Table 2). We did not detect IgG against *A. phagocytophilum*.

## Conclusions

We report a confirmed case of *Candidatus* N. mikurensis infection in Spain, detected in human bone marrow aspirate, serum, and EDTA-blood samples, that was no longer detected months after completing antimicrobial drug treatment. A broad clinical spectrum of tickborne diseases is found in Spain. Human cases of Lyme borreliosis, Mediterranean spotted fever, and other tickborne rickettsioses have been described, including *Dermacentor* tick–borne necrosis erythema lymphadenopathy, *Rickettsia sibirica mongolitimonae* infection, *R*. *massiliae* infection, *R*. *aeschlimannii* infection, babesiosis, human anaplasmosis, tularemia, *Borrelia hispanica* relapsing fever, tick paralysis, Crimean-Congo hemorrhagic fever, and α-gal syndrome or other allergic reactions (*14*). Since we discovered *Candidatus* N. mikurensis in *I. ricinus* ticks in Spain (*13*), we have conducted surveillance of this bacterium. *Candidatus* N. mikurensis should be considered a potential cause of persistent fever and venous thrombosis in patients with hematologic malignancies who live in environments where ticks are prevalent. *Candidatus* N. mikurensis infections should be particularly suspected in patients who are immunosuppressed but also should be considered in patients with other vascular conditions who are not immunocompromised (*15*).
